# Supplementary figures and images for: The movements of a recently urbanized wading bird reveal changes in season timing and length related to resource use
Source: PLoS One. 2020 Mar 19;15(3):e0230158. doi: 10.1371/journal.pone.0230158 (PMC7082014; doi:10.1371/journal.pone.0230158)

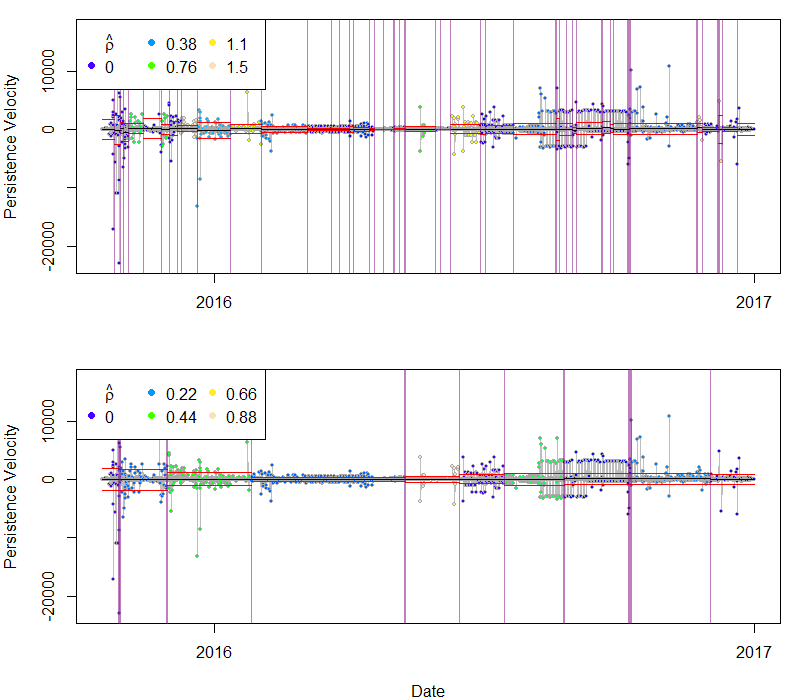

Supplement: S1 Fig — The persistence velocity (y-axis) between consecutive locations is calculated with the BCPA and plotted over time. Vertical lines indicate the significant change points with the width of the lines proportional to the number of times that change point was selected in the moving window analysis. The black and red lines show the mean and standard deviation estimate of the persistence velocity. The coloured circles (ρ hat in the legend) reflect the autocorrelation time scale (Gurarie 2013). Upper panel shows the unfiltered BCPA output depicting every change point selected in the moving window analysis. Lower panel shows the filtered BCPA output that selects significant change points from the neighbouring change points within 10 days. (TIF) [file pone.0230158.s001.tif]

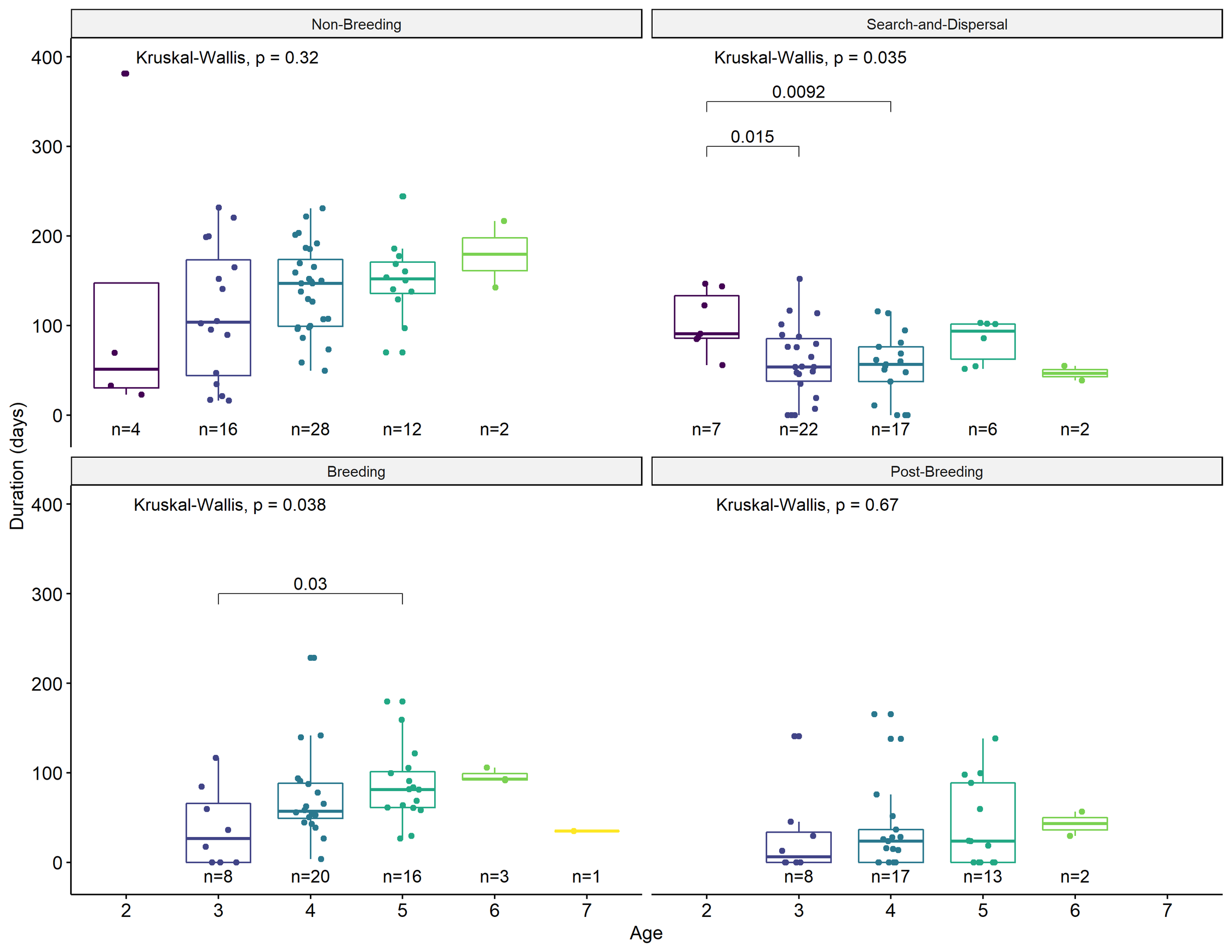

Supplement: S2 Fig — Distribution of number of days in each behavioral season (non-breeding, search-and-dispersal, breeding attempt, and post breeding) according to ibis age. (TIF) [file pone.0230158.s002.tif]

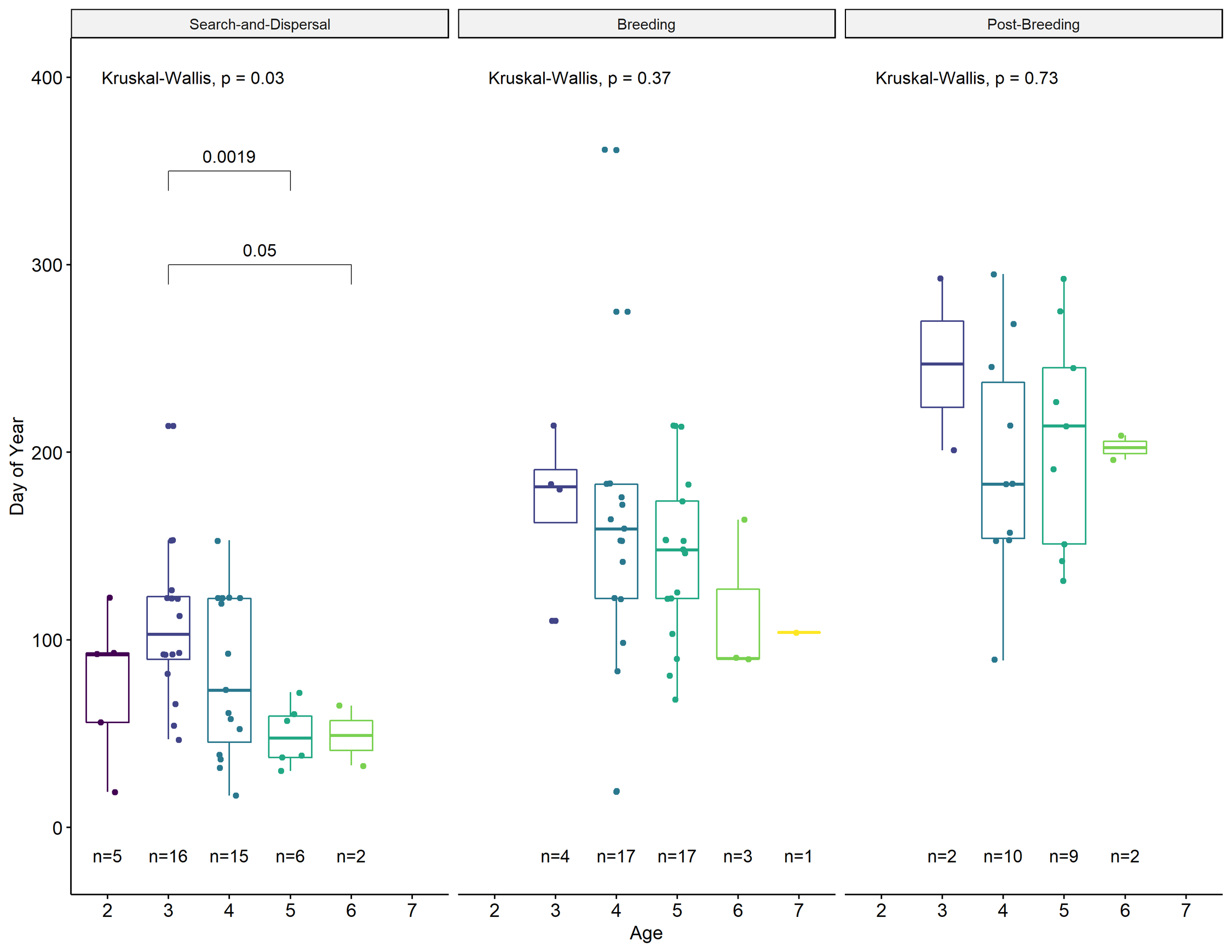

Supplement: S3 Fig — Distribution of number of days in each behavioral season (search-and-dispersal, breeding attempt, and post breeding) according to ibis age. (TIF) [file pone.0230158.s003.tif]

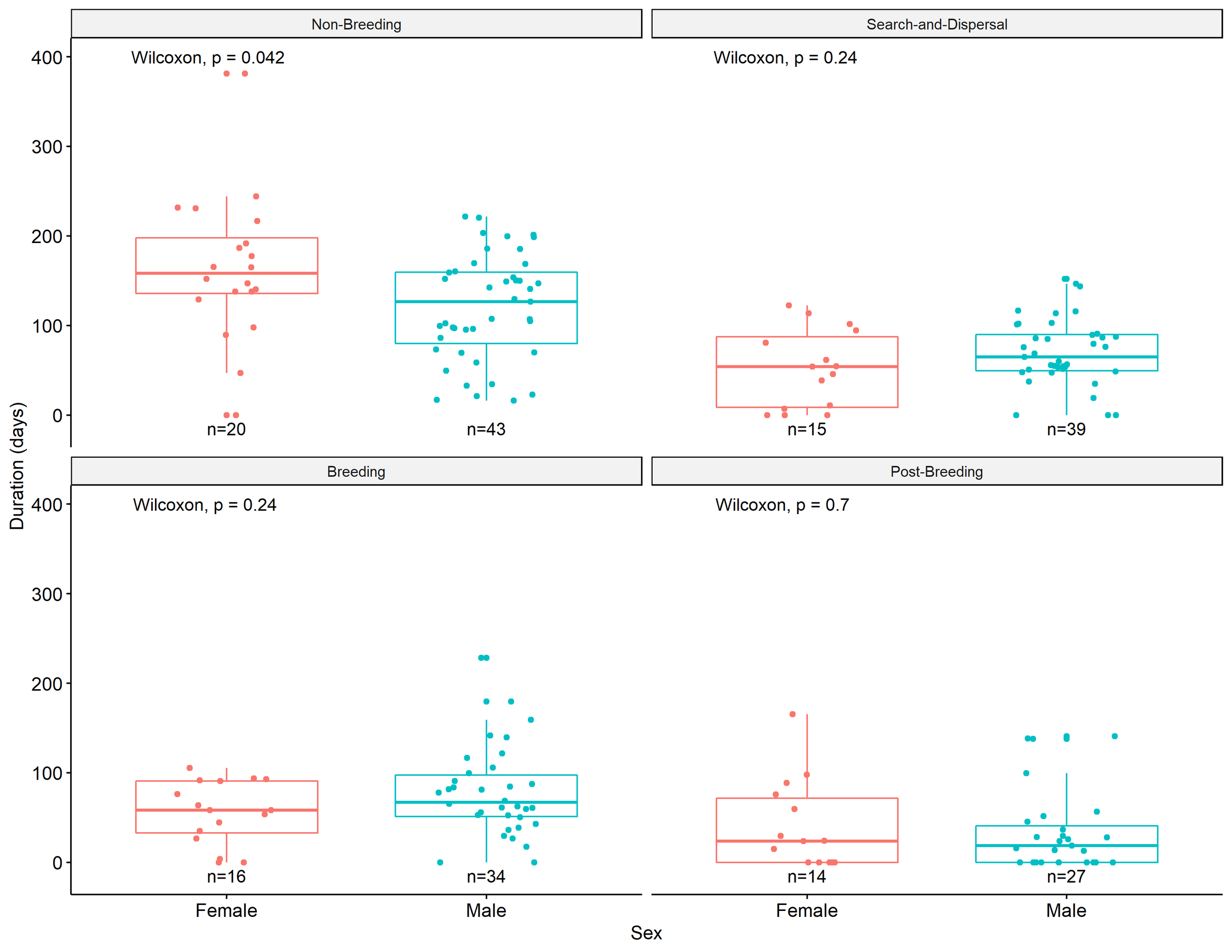

Supplement: S4 Fig — Distribution of number of days in each behavioral season (non-breeding, search-and-dispersal, breeding attempt, and post breeding). (TIF) [file pone.0230158.s004.tif]

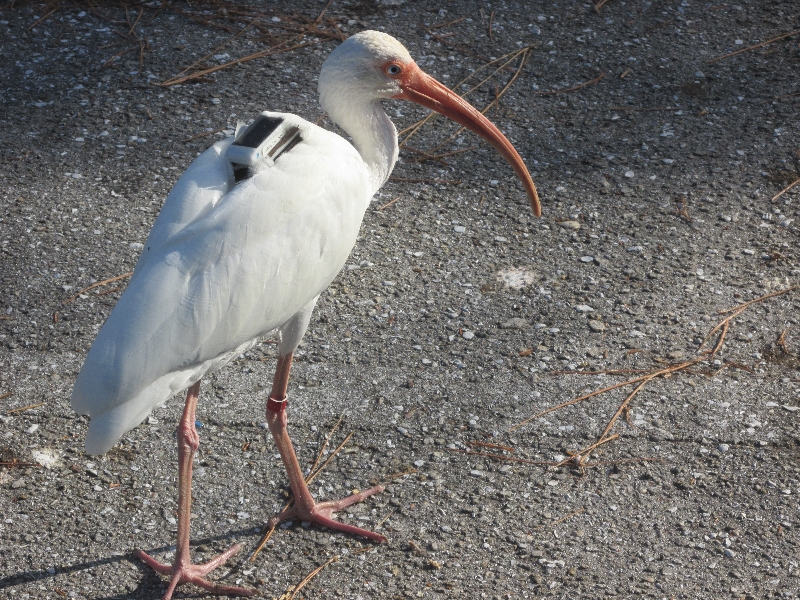

Supplement: S5 Fig — (JPG) [file pone.0230158.s005.JPG]
