# Supplementary material for: The movements of a recently urbanized wading bird reveal changes in season timing and length related to resource use
Source: PLoS One. 2020 Mar 19;15(3):e0230158. doi: 10.1371/journal.pone.0230158 (PMC7082014; doi:10.1371/journal.pone.0230158)
Supplement: S1 File — (PDF) [file pone.0230158.s006.pdf]

S1 File. Defining ibis BCPA bouts as behavioral seasons

We acknowledge that we cannot be certain what exactly an individual is doing at any point in time because we only have animal locations and lack “on the ground” information. However, we can examine the pattern of locations on the landscape and compare them to known patterns of ibis behaviors throughout their annual cycle to hypothesize in which behavioral state they may be. We used a decision tree (Figure 2) to classify bouts into behaviors based on the land cover under locations and the spatial pattern of locations during the bout.

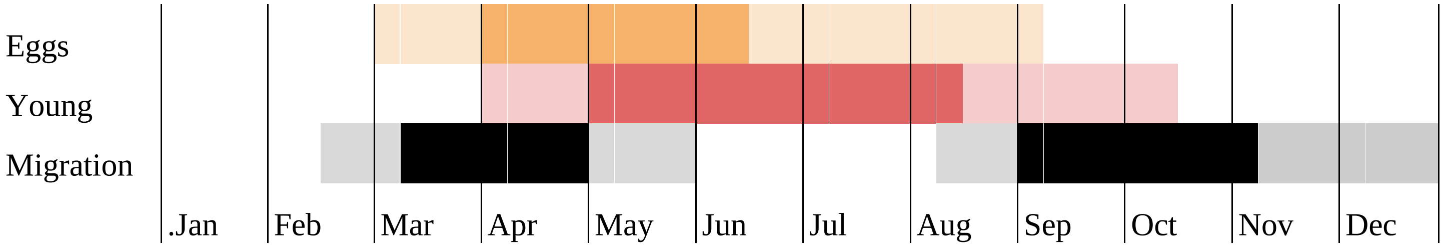

Figure 1: Redesigned annual cycle for White Ibis from Birds of North America Article.

To begin classifying BCPA bouts into defined behavioral seasons, we first selected a bout that occurs during the predicted “Eggs” or “Young” season according to the White Ibis article in Birds of North America (Figure 1). Breeding attempts should be the easiest to identify because they are defined by very specific resource requirements. As nesting birds, breeding attempts should also be characterized by repeated visits to nest sites, which would be visualized by dense aggregations as a single location.

As stated above, we acknowledge that we cannot be certain if an individual is actively breeding at any point in time, or if their breeding attempts are successful, because we only have animal locations and lack “on the ground” information. However, we use the pattern of locations to look for patterns that indicate a breeding attempt may be occurring. Breeding attempts were characterized by: 1) occurring within the eggs and young window, highlighted in orange and red (Figure 1), 2) a dense aggregation of locations in a wetland area or near a known rookery site (e.g., SWA rookery, PGA rookery, LWR boat ramp rookery or interior rookeries), 3) lasting on average 10 weeks, but previous studies have found breeding activities to last from 6 weeks to 6 months. When characterizing breeding attempts we note that shorter bouts could occur where there is a failed breeding attempt and every day might not be spent at the hypothesized rookery site. Male and Female ibis share nesting and rearing responsibilities and, thus, parents can spent multiple days far away from the rookery foraging and staying at a separate roost site.

Non-breeding and “migratory periods can be difficult to distinguish and might require comparing to other bouts. There are 2 “migratory” periods for ibis: pre-breeding and post-breeding. Ibis are not true migratory animals, so these periods might or might not be displayed. The pattern of locations for migratory and non-breeding behavioral season can also take on different characteristics depending on the bird’s behavior. We will call the pre-breeding period a “Search or Dispersal” phase as in individual relocates to or looks for an appropriate rookery site and mate. The “Post-breeding” phase could be exhibited as a bout in which and animal relocates from the breeding range to the non-breeding range, or begins to resume nomadic movements. When ibis are not breeding or making breeding related movements, their behaviors are least constrained, meaning that they can make foraging and habitat selections for their own survival without the concerns of foraging for young or selecting nest sites. Non-breeding seasons could be exhibited as nomadic movements between several areas or residential movements around one or more reliable foraging areas.

For migratory periods we look for patterns that are characterized by 1) linear-like movement as an individual moves its range from one place to another or 2) a range expansion, or movement beyond the typical range.

Search-and-dispersal or post-breeding behaviors could be indicative of birds moving between 2 distinct ranges, or evidence of birds exploring potential areas for breeding before or after a nesting attempt.

Non-breeding seasons are characterized by: 1) residential movements in which an ibis consistently uses a few locations that typically include a park or residential area where they find reliable food and a consistently used roost. Residential non-breeding seasons have a clear activity center and consistently used roosts. 2) local nomadic movements in with ibis use several daytime locations within a small area. There might or might not be a clear activity center, but there are consistently used roosts. 3) nomadic movements in which ibis move between several roosts and foraging areas without a clear activity center and there might not be consistently used roosts.

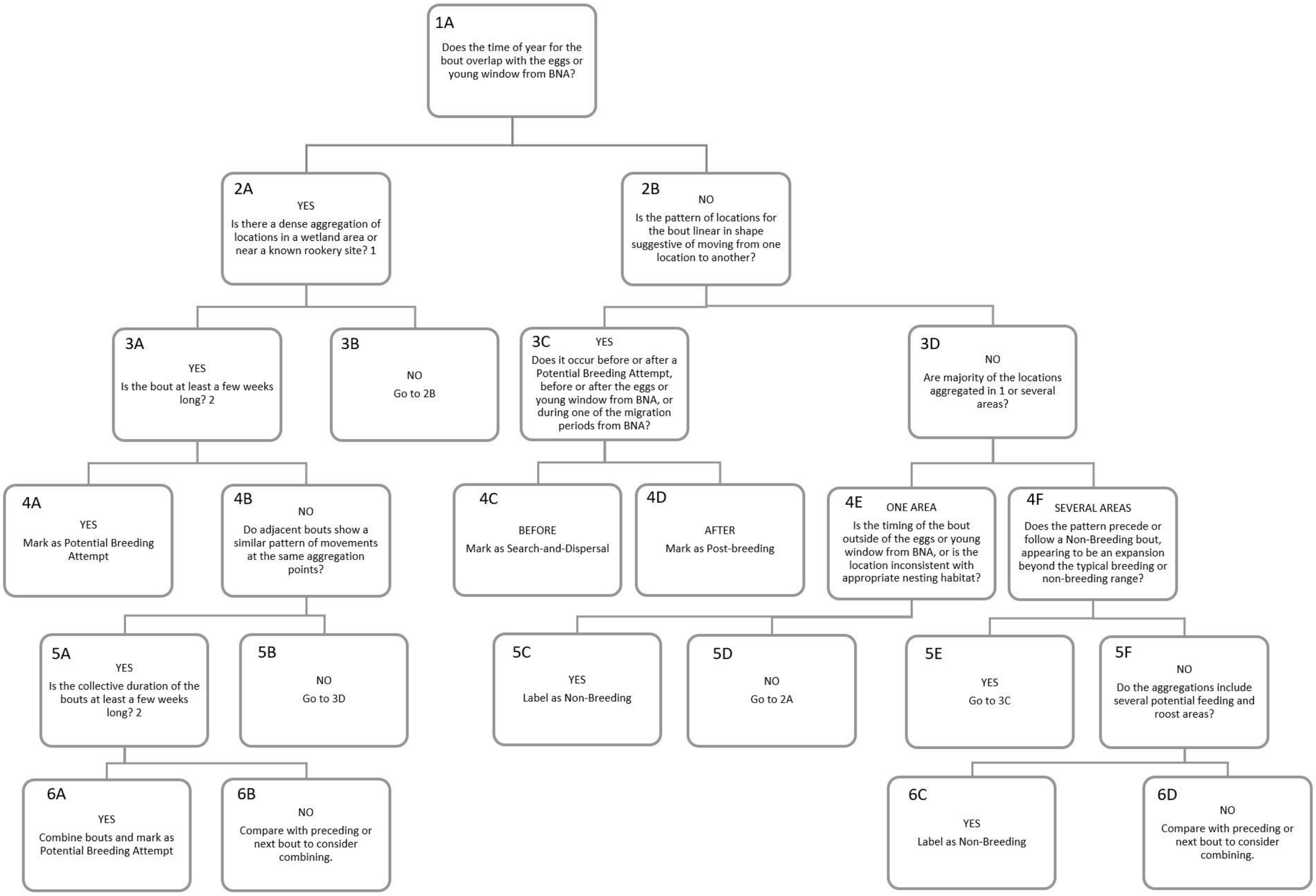

1: parents may not spend every day at the rookery site. Parents may spend multiple days at an alternate roost site. Males and Females co-parent.

2: successful breeding takes on average 10 weeks, but can take as little as 6; less time could indicate a nesting failure

Figure 2: Flow chart used to assign behavioral season names to specific bouts of similar behavior identified by behavioral change point analysis.
