# Supplementary material for: The movements of a recently urbanized wading bird reveal changes in season timing and length related to resource use
Source: PLoS One. 2020 Mar 19;15(3):e0230158. doi: 10.1371/journal.pone.0230158 (PMC7082014; doi:10.1371/journal.pone.0230158)
Supplement: Attachment — Submitted filename: Response-to-Reviewers.docx [file pone.0230158.s007.docx]

PONE-D-19-24467
The movements of a recently urbanized wading bird reveal changes in season timing and length related to resource use
PLOS ONE

Dear Dr. Hepinstall-Cymerman,

Thank you for submitting your manuscript to PLOS ONE. After careful consideration, we feel that it has merit but does not fully meet PLOS ONE’s publication criteria as it currently stands. Therefore, we invite you to submit a revised version of the manuscript that addresses the points raised during the review process.

Academic Editor

The two reviewers raise a number of important issues that need to be addressed before this manuscript can be considered for publication in PlosOne.  I therefore invite you to make major revisions to the manuscript, in the light of the reviewers’ comments.  Please pay close attention to the major issues raised by Reviewer 2.

All points raised by the reviewers must be addressed in the revised manuscript, giving page lines of changes for easy reference, or as rebuttals in an accompanying letter.

Journal Requirements:
1. When submitting your revision, we need you to address these additional requirements. Please ensure that your manuscript meets PLOS ONE's style requirements

*Response: We believe we have checked and fixed as needed all issues related to this request.*

2.  We note that Figure1 in your submission contains map images which may be copyrighted. All PLOS content is published under the Creative Commons Attribution License (CC BY 4.0), which means that the manuscript, images, and Supporting Information files will be freely available online, and any third party is permitted to access, download, copy, distribute, and use these materials in any way, even commercially, with proper attribution. For these reasons, we cannot publish previously copyrighted maps or satellite images created using proprietary data, such as Google software (Google Maps, Street View, and Earth). For more information, see our copyright guidelines: <http://journals.plos.org/plosone/s/licenses-and-copyright>. We require you to either (1) present written permission from the copyright holder to publish these figures specifically under the CC BY 4.0 license, or (2) remove the figures from your submission:

*Response: We have removed the ArcGIS basemap images and replaced them with imagery from the USGS National map, specifically from the National Agricultural Imagery Program, which are not copywritten.*

In your Data Availability statement, you have not specified where the minimal data set underlying the results described in your manuscript can be found. PLOS defines a study's minimal data set as the underlying data used to reach the conclusions drawn in the manuscript and any additional data required to replicate the reported study findings in their entirety. All PLOS journals require that the minimal data set be made fully available. For more information about our data policy, please see http://journals.plos.org/plosone/s/data-availability.

 Upon re-submitting your revised manuscript, please upload your study’s minimal underlying data set as either Supporting Information files or to a stable, public repository and include the relevant URLs, DOIs, or accession numbers within your revised cover letter. For a list of acceptable repositories, please see http://journals.plos.org/plosone/s/data-availability#loc-recommended-repositories. Any potentially identifying patient information must be fully anonymized.

 Response: *We have created the required public repository as part of Movebank. The reference is: Kidd-Weaver A, Hepinstall-Cymerman J, Welch CC, Murray MH, Adams HC, Ellison TJ, Yabsley MJ, Hernandez SM (2020) Data from: The movements of a recently urbanized wading bird reveal changes in season timing and length related to resource use. Movebank Data Repository.* [*https://doi.org/10.5441/001/1.8ms50757*](https://doi.org/10.5441/001/1.8ms50757) *and will be made available if and when the article has been accepted for publication and published by PLOS.*

Additional Editor Comments (if provided):

Academic Editor
The two reviewers raise a number of important issues that need to be addressed before this manuscript can be considered for publication in PlosOne. I therefore invite you to make major revisions to the manuscript, in the light of the reviewers’ comments. Please pay close attention to the major issues raised by Reviewer 2.
All points raised by the reviewers must be addressed in the revised manuscript, giving page lines of changes for easy reference, or as rebuttals in an accompanying letter.

*Response: We believe we have addressed each specific comment by both reviewers as well as the general overarching comments. We provide responses to each comment below.*

[Note: HTML markup is below. Please do not edit.]

Reviewers' comments:

Reviewer's Responses to Questions

**Comments to the Author**

1. Is the manuscript technically sound, and do the data support the conclusions?

The manuscript must describe a technically sound piece of scientific research with data that supports the conclusions. Experiments must have been conducted rigorously, with appropriate controls, replication, and sample sizes. The conclusions must be drawn appropriately based on the data presented.

Reviewer #1: Partly

Reviewer #2: No

2. Has the statistical analysis been performed appropriately and rigorously?

Reviewer #1: Yes

Reviewer #2: I Don't Know

3. Have the authors made all data underlying the findings in their manuscript fully available?

The [PLOS Data policy](http://www.plosone.org/static/policies.action#sharing) requires authors to make all data underlying the findings described in their manuscript fully available without restriction, with rare exception (please refer to the Data Availability Statement in the manuscript PDF file). The data should be provided as part of the manuscript or its supporting information, or deposited to a public repository. For example, in addition to summary statistics, the data points behind means, medians and variance measures should be available. If there are restrictions on publicly sharing data—e.g. participant privacy or use of data from a third party—those must be specified.

Reviewer #1: Yes

Reviewer #2: Yes

4. Is the manuscript presented in an intelligible fashion and written in standard English?

PLOS ONE does not copyedit accepted manuscripts, so the language in submitted articles must be clear, correct, and unambiguous. Any typographical or grammatical errors should be corrected at revision, so please note any specific errors here.

Reviewer #1: Yes

Reviewer #2: Yes

5. Review Comments to the Author

Please use the space provided to explain your answers to the questions above. You may also include additional comments for the author, including concerns about dual publication, research ethics, or publication ethics. (Please upload your review as an attachment if it exceeds 20,000 characters)

Reviewer #1: General comments

This is an interesting study with a large sample of GPS tracked individuals. The framing of the study is interesting, assessing seasonal behaviors with respect to underlying habitat use as defined by human modification of the land. I believe the study makes a nice contribution to our understanding of animal behavior in response to human land use modification. However, revisions are required to improve the clarity of the results. Many sentences in the results focus on “urban habitat use” however this phrasing often lacks clarity.

*Response: We have attempted to clarify this concept when we first use it and throughout the remainder of the manuscript. The concept, “individual ibis use of urban habitats during the non-breeding season” is quite a long term. We have reworded “urban habitat use” to “use of urban habitat(s)” throughout the manuscript as well as a more complete introduction to the specifics of the measure we used to quantify this concept in the Methods.*

Abstract

Line 23: edit “behavioural” to “behavioral” or vice versa throughout.

*Response: We have changed to “behavioral” throughout*

Introduction

Line 59 & 62: edit “behavioural” to “behavioral” or vice versa throughout. I’ll not flag all of these, they occur throughout the document.

*Response: We have changed to “behavioral” throughout the manuscript, tables, and captions.*


Line 66-67: suggested edit “…the behavioural state of an individual rather than an arbitrary calendar date…” to “…the behavioral state of individuals, representing the “population”, rather than an arbitrary calendar date…”

*Response: We have reworked this section to read as follows: “The behavioral state of individuals from a local population is inferred from their movement patterns rather than an arbitrary calendar date or highly variable biotic signals.” And in response to Reviewer 2’s comments below, we have added a second sentence with an example, “For example, an animal may exhibit high levels of tortuosity while attending a nest or while using the same resource repeatedly, exhibit linear movements between disparate ranges, or exhibit exploratory movements that take on a nomadic pattern."*

Line 66-67: suggested edit “Determining behavioural seasons based on animal movement data is a critical need to advance the understanding movement ecology of animals” to “Determining behavioural seasons, based on animal movement data, is critically needed to advance the understanding the movement ecology of animals” or you could go further: “Determining biological meaningful seasons, based on animal behavior, is needed to advance our understanding of animals movement ecology”.

*Response: Reworded to, “Determining behavioral seasons, based on animal behavior, is critically needed to advance our understanding of movement ecology of animals”*

Line 77-78: What is meant by “urban habitat used” in this sentence: “We predicted that ibis are changing when and where they move related to the amount of urban habitat used.” Do the authors mean “urban habitat modified by humans or does this relate to ibis mobility?” Please clarify.

*Response: Reworded to, “We predicted that ibis are changing their movement patterns as a direct result of their experience with and use of urban habitats.”*

Methods

Line 110: edit to past tense “In recent decades, ibis living in southern Florida are increasing their use of…” to “In recent decades, ibis living in southern Florida have increased their use of…”.

*Response: Changed as suggested.*

Line 120: the abbreviation “FL” was not been provided earlier, expand here or add in previous section “Palm Beach County, FL”.

*Response: Changed to Florida.*


Line 200: edit “separating migratory segments of animal migrations” to “separating segments of animal migrations”.

*Response: Changed as suggested.*

Results

The first paragraph of the results section is mostly methods, I suggest moving the whole paragraph or most of the detail to the methods.

*Response: We have moved this paragraph to the Methods under the” Ibis capture…” heading*

All Results section: expand the abbreviated seasons SD, NB, BR, PB as they aren’t used that often and spelling the words is much clearer.

*Response: Changes made throughout manuscript, figures and tables and their captions.*

Lines 252-255. What does n = 62, n = 54, n = 48 and n = 40 refer to? N = 48 birds were tracked, n = 41 were included in the analyses.

*Response: ‘n’ here refers to the number of seasons for each of the four seasons and because we had multiple years of data for some birds, the ‘n’ for each season will be larger than the ‘n’ for individual birds (41). We added the following sentences to the start of that paragraph to explain the discrepancies in numbers. “We classified each individual ibis timeline into seasons and performed analyses based on the seasons exhibited by individuals, the duration of each season, and the initiation dates for each season. The number of seasons recorded is not equal to the number of individual birds tracked because ibis had variable timelines (e.g., some birds were tracked through multiple non-breeding seasons while others were only tracked through one).”*

Lines 252-281: This section refers to Figure 2, this figure contains a lot of information which isn’t automatically clear. Clarify that juveniles/sub-adults were “adults” when included with respect to breeding season. I suggest you break this figure into a panel of 6 figures: separate birds by sex (e.g. males on the left females on the right), with individual figures for “low”, “intermediate” and “high” urban ibis use.

*Response: We explored several different ways of representing these data and retained the single panel, but did not feel other representations were more effective. This figure is complex, but it is attempting to display many ideas at once in a consolidated manner. We have rotated the figure 90 degrees clockwise believing that slight change would improve reader comprehension. We have also modified the figure caption to add additional details. Because this figure is also sorted by the amount of non-breeding season daytime urban habitat use, separating the figure into multiple panels or sorting on different items would remove this information. Additionally, in response to this comment and others by the other reviewer, we also clarified in the manuscript text the rationale behind classification of juvenile or sub-adult seasons into “breeding” even though it was unlikely that these specific birds were producing offspring.*

Lines 286-289: the first part of this sentence reports a result “Ibis daytime use of urban habitat classes in the NB season ranged from 0.3% to 68.6%...” however, the second part is methods and should be moved “which, to facilitate statistical comparisons, we classified into three quantiles defined by cut points at 28.2% and 54.9% representing “low”, “intermediate”, and “high” urban-use.”

*Response: We have moved this information to the last paragraph of the Methods.*

Lines 289-290: the focus of this paper is “seasonal behavior”, this sentence lacks clarity and doesn’t include an assessment of “season”. Given the various timing of deployment, deployment periods, locations and bird age this statement requires further detail to explain what exactly is being tested. “Urban and wetland habitat use were negatively correlated (Pearson Correlation = -0.730).”

*Response: We moved this text to the Methods, and reworded this section to say, “To address our goal of exploring how individual association with urban environments may predict changes in the timing and duration of behavioral seasons, we grouped ibis by their daytime use of urban land cover/land use classes (hereafter “urban habitats”) in the non-breeding season into quantiles. We then compared the amount of time (duration) individuals spent in each defined behavioral season and the initiation date of each season to identify relationships relative to the use of urban habitat of a given individual during the non-breeding season. Use of urban habitats in the non-breeding season ranged from 0.3% to 68.6% and were subsequently grouped into three classes by quantile: 0-28.2%, 28.2-54.9% and >54.9%, representing “low”, “intermediate”, and “high” urban-use, respectively. We tested for differences in season duration and initiation for interactions between sex and age or sex and urban use class using Kruskall-Wallis multiple comparison tests, Two-Sample Wilcoxon Tests, as appropriate detailed in the previous section. Comparisons were made for all seasons except for initiation date of the non-breeding season because its start timing was highly dependent on the timing of other seasons.*

*We also removed the sentence comparing urban and wetland habitat use as that was not the focus of this paper.*

Figure’s 3, 4 & 5. The points along the x-axis, within each category (e.g. land use, age), are distributed with respect to an undefined “period”. The x-axis label doesn’t contain enough information for the reader to understand how points within each category vary. Please clarify.

*Response: Individual points were included in each boxplot using “jitter” with ggboxplot in R. This information has now been included in figure captions.*

Figure 3. Presumably birds of all ages are included, however juvenile and sub-adults wouldn’t engage in breeding activity, please clarify that these birds have not been included where they were immature.

*Response: This assumption is incorrect. Birds do move to breeding colonies and engage in observation of other’s nesting efforts or to engage in direct helping of active nests (Bildstein, 1993, Page 85). While they may not be actively breeding themselves, they are exhibiting movement behaviors that are consistent with a breeding season. Without ground truth information about what exactly birds are doing at any point in time, we are restricted to our assumptions based on the behavioral patterns defined in our analysis. Also, ibis researcher Peter Fredrick (personal communication) has noted in his captive colony work that 3 year old ibis, which we refer to as subadults, are capable of breeding. In our data, we did not find any breeding seasons for juvenile ibis.*

Figure 3. How are birds “grouped by daytime, non-breeding season urban habitat use classes” when this figure assesses all “Four behavioural seasons (non-breeding, search-and-dispersal, breeding attempt, and post breeding)”?

*Response: We believe the reviewer missed the section in Results where we grouped individual birds by their non-breeding season daytime use of urban habitats. We have reworded that section in the Results (see above). Figure 3 compares the duration of each individual’s respective season grouped by* *daytime use of urban habitats during the non-breeding behavioral season. We reworded the figure caption to better convey this information.*

Lines 308-310: suggested edit to increase clarity (similar edits could be made throughout the results. “Significant differences in season initiation dates by urban habitat use differed only in the BR season (Fig 4) such that ibis with high urban-use began their BR seasons earlier in the year than those with low urban-use.” Revised “Ibis with greater use of urban habitats began their breeding season earlier than ibis with lower use of urban habitats (Fig 4).” Many sentences focus on “urban habitat use” however this phrasing often lacks clarity.

*Response: We have added details here and elsewhere on what we were measuring and why and changed abbreviated wording to “use of habitat” throughout.*

Figure 4. Delete juveniles, their behavior does not relate to “breeding season” and shouldn’t be assessed in this framework, they are immature.

*Response: We respectfully disagree with this comment. While juveniles may not breed, they do exhibit a change in movement behavior and may explore nesting colonies and do exhibit search-and-dispersal behavior. In Bildstein’s book, “White Ibis”, he noted “several dozen to several hundred nonbreeding, second-year, subadult White Ibises, who … spend a significant amount of time attending to nestlings whose parents are off foraging.” (pg 85)*

*The current format of Figure 4 clearly shows no juveniles in the breeding and post-breeding seasons. Separating this figure into two figures to have one that does not include juveniles seems inefficient and potentially more confusing. We have left this figure as it was originally.*

Lines 313-315: The 2018 sample size is questionably low for inclusion in this analysis, I suggest removing it where it is <3 and the authors consider removing 2018 from these analyses all together. “Yearly differences in initiation dates were significantly different for intermediate urban-use ibis only in the SD season, which started significantly later in 2016 than either 2017 or 2018 (Fig 5). For high-urban use ibis, initiation dates differed significantly in both the SD and BR seasons, both starting later in 2016 than 2017 or 2018.”

*Response: In 2018 we still had 6-7 bird sending us their locations. We acknowledge that 6 is still a small sample size, it is indicative of patterns.*

Lines 332-337: lack clarity, revise to statements.

*Response: We revised this section for clarity.*

Lines 340-341: As breeding requires birds to be sexually mature this finding goes without saying. If you are testing adult ibis age then this is meaningful, however the methods do not describe a way to age adult ibis other than to say they are adults. Furthermore, figure 5 does not present age at all. If you are referring to figure 4 then adults are lumped together and compared to juveniles and sub-adults, which does not support this sentence. “Older ibis tended to spend more time dedicated to breeding with mean season duration by age significantly different for SD and BR seasons (Fig 5).”

*Response: Our mistake in uploading the wrong figure here. We elected to remove the figure that shows season duration by year-specific ages. We have elected to reintroduce this figure as a supplementary figure. We have also removed this subsection and provided a summarized set of results by age or sex in the “Behavioral season timelines” section with supporting figures moved to Supplemental Materials.*

Lines 341-343: I question the value of this result as “juveniles” were not sexually mature and would not engage in breeding activities. “Juveniles had significantly longer SD seasons compared to both subadults (with 38% subadults (n=8) skipping this season altogether) and adults.”

*Response: As mentioned in previous responses, juveniles and subadults might exhibit the behaviors associated with looking for a place to breed or attempting to engage in breeding activities. We believe that there is still value in these conclusions to indicate that, though sexually immature, ibis of all ages still alter their behaviors throughout the year. We have reframed our results to focus more on the results that all ibis switch between multiple behavioral states rather than the difference between older and younger ibis.*

Lines 343-345: is this biologically meaningful, did sub-adults engage in breeding? “Adults had longer breeding seasons than subadults. Season initiation dates were suggestive of differences in the SD season (p < 0.1) with subadults starting later in the year than adults.” If sub-adults engaged in breeding does that mean they transitioned to be “adults” and thus comparing them with “sub-adults” isn’t possible?

*Response: Observations of ibis in other studies have indicated that subadult ibis might be capable of breeding, but typically do not in the wild. Also, they might exhibit movements while observing active nests and rookeries, or might act as “helpers” as mentioned in responses above, that are indistinguishable from breeding behavior based on tracking data alone.*

Lines 345-349: this sentence lacks clarity, revise to statements “Seasons skipped also varied with age: three each of the six skipped SD seasons were skipped by subadults (14% of subadult SD seasons skipped) and adults (12%); 3 of skipped breeding seasons were by subadults (38%); 4 of the 14 skipped PB seasons were by subadults (50%), and 10 were skipped by adults 4 (31%).”

*Response: We have elected to remove these sentences as they do not add much detail to our discussion.*

Lines 355-358: lack clarity, revise to statements.

*Response: We have elected to remove these sentences regarding differences by sex as they do not add much detail to our discussion and consolidate abbreviated reporting of differences in duration or initiation date of behavioral season by age or sex differences to the first section of the Results, Behavioral season timelines.*


Discussion

The Discussion should be revised with respect to the comments on the results e.g. Lines 365-367: consider removing reference to “age” – “We found that season initiation and duration are related to urban habitat use, individual traits such as age and sex, and annual variation likely related to yearly environmental variations.”

*Response: We have retained discussion of age as it was clear the ibis changed their movement behavior between seasons, but we have reframed the discussion to be more clear why it is a valid analysis. We have reworded this sentence as follow, “We found that season initiation and duration are related to urban habitat use, modulated by individual traits such as age and annual variation likely related to yearly environmental variations”.*

Lines 375-379: If this point relates to the time spent by adult vs non-adults then it needs to be revised as this isn’t a valid comparison. “We found the amount of time spent in pre-breeding and breeding seasons shifts with age; older birds spend less time looking for breeding sites and more time exhibiting reproductive behaviours, likely related to increased experience as ibis learn how to better locate rookeries and raise successful clutches as they age.”

*Response: We have retained these analysis and this discussion as it is relevant. See responses to comments related to the Methods and Results above.*

Lines 379-382: I’ve been unable to revise the original reference, however I question the stated behavior of “younger ibis serving as helpers”. Have the authors observed this behavior directly associated with “cooperative breeding” as is implied by this sentence? “For younger ibis, longer search-and-dispersal seasons, especially in the absence of breeding seasons, are likely evidence of ibis serving as helpers in colonies to gain nesting experience before their own breeding attempts [19].”

*Response: We have not observed this behavior ourselves, but other studies have documented this (Reference #19, K.L. Bildstein book, “White Ibis: Wetland Wanderer”, page 85)*

Line 402: as stated above, this sentence may need to be revised – “We found changes in seasonal timing and duration related to age.”

*Response: We have changed the order and focus of the discussion to reflect change in focus of the results.*


Reviewer #2: I appreciate the time and effort that went into this work but I'm afraid that the manuscript has two major flaws that significantly limit the research. First, the conceptual basis, justification, and objectives of the study are not at all clear. The meaning of "behavioral season" in the Introduction as it relates to movement left me wondering whether all types of movement were being considered or just what one typically thinks of as seasonal movement, i.e. dispersal and migration. This is cleared up in the Methods but needs to be explained much better up front, in the Introduction.

*Response: We have rewritten the introduction to make this focus clearer. Responses to specific line comments were made above in our responses to Reviewer 1. We have analyzed all points to look at two specific movement metrics to find changes in movement patterns, which were then used to define changes in behavioral seasons relating to changes in behaviors associated with each season (searching for a breeding site, breeding, searching for new roosting and feeding sites after breeding, resuming nomadic behaviors associated with following ephemeral resources across the landscape). We debated moving the entire “Study System” subsection from Methods to the Introduction, but have left the organization as is, with insertion of the statement: “Ibis in this region move to follow ephemerally available food resources and generally exhibit four identifiable types of movements associated with different seasons (details under Study System below)” just prior to the objectives statement.*

The objectives of the study are to define behavioral seasons and to understand how behavioral seasons vary with respect to habitat type (wild vs urban). The justification for the first objective eludes me. How does identifying individual season start and end dates help us? Of course, season duration and timing will vary among individuals. This is a basic tenet of niche ecology and the data presented in this manuscript exemplify it well. On the other hand, using environmental cues to define seasons is useful. Environmental cues vary regionally and researchers are thereby not misled by date. This is the approach used by large-scale citizen science programs and works well. If the purpose of the study is to avoid the problems described at lines 444-446, then this is useful but the study then becomes a methodological one and the Introduction should be framed accordingly.

*Response: We are using the movement data of the birds to define when they are exhibiting movement behaviors consistent with a particular season. Florida lacks clear phenological cues to seasons, however in natural settings ibis respond to water levels, searching for and exploiting resources associated with water depths suited for ibis bill and leg length. Using observed movements to define seasons and comparing timing of seasons and season duration is the focus of the second objective, which is dependent on defining seasons based on movements. To make this clearer, we have reworded our objective statement as follows: “The objective of our study was to determine if and how white ibis in this area change their annual seasonal expression based on experience with wetland and urban land uses. Specifically, we predicted that the timing of seasons (season initiation) and length of each season (season duration) would differ with the degree of urban habitat association.”*

The justification for the second objective is just as unclear. What do we know about urbanization and the timing and duration of species phenology as it relates to birds? Quite a bit, including what is described at lines 112-114. What does this study add to existing knowledge? Earlier onset of breeding has been demonstrated for many species in urban areas. The shorter dispersal period of ibis in urban habitats in the study area is interesting and potentially novel, but again, the authors should re-frame the paper to make this more of a focus if that is the intent.

*Response: We reframed the Introduction to make it more clear that the focus of this study was to explore how non-breeding season urban habitat use changes seasonal timing and duration. Observed breeding for urban-using Ibis was NOT earlier as these birds had to move from urban areas to more natural areas to find suitable breeding locations (rookeries). We did observe birds moving to breeding locations only to return the next day/within a few days, likely because breeding conditions were not suitable or con-specifics were not yet present, only to return to the same rookery weeks later and stay for multiple week.s*

Second, I'm not convinced that the study design is adequate to address the objectives. It appears that different birds were tracked during different seasons and years. It would be ideal to track multiple individuals throughout all seasons in all years (or over one year) to avoid the possibility that results in one season, ie season duration, are driven by a few idiosyncratic individuals. It's not at all clear whether individual is the correct unit of analysis to analyze the effect of habitat type on season duration and timing. Do individuals within a site influence each others' movement? Finally, how does inter-annual variation affect the results? Are habitat type and year confounded? Some of these concerns may be unfounded but I found it very difficult to determine if this was the case.

*Response: We have revised the methods, results, and discussion to be more clear on what we did, what we found, and how we interpret these findings. Yes, each bird has a different set of data available, some with multiple years, others with less data (See figure 2 which shows the deployment date for each bird [the left boundary] as well as the timeline of data included [the right boundary]. Once seasons were defined for each individual (for one or more years), the initiation date and duration was extracted for each unique bird-season. We did censure data for birds with not enough data to define at least one year, and tested for year differences because of changes in phenology and environmental cues between years (i.e., more or less precipitation in ~February-March will affect the suitability of rookery sites later in the spring). These animals are social feeders and group roosters/nesters, so what our animals did should be generally representative of what other individuals are doing. It is unclear if the concept of “population” is even an appropriate concept for these animals at this time in this region given the apparent mixing and movement between urban and wetland areas and is beyond the scope of this paper.*

*Finally, we did indeed find an effect of year, primarily on season initiation because water levels dictate when breeding locations are safe (hold water surrounding a central breeding tree(s)) to breed.*

Two more minor comments: 1. The description of the definition of behavioral seasons at lines 216-224 seems very objective. I have a hard time seeing someone else being able to replicate these methods. Does this significantly impair the utility of the behavioral season concept?;

*Response: We have provided the specific decision rules the lead author used when making these classifications as supplemental materials.*

2. The Discussion section is mostly inference that is very weakly based on the results. Too much is being inferred from the results as they stand.

*Response: We have reduced and focused the discussion.*
